# Supplementary figures and images for: A study to assess current approaches of allergists in European countries diagnosing and managing children and adolescents with peanut allergy
Source: PLoS One. 2020 Dec 3;15(12):e0241648. doi: 10.1371/journal.pone.0241648 (PMC7714149; doi:10.1371/journal.pone.0241648)

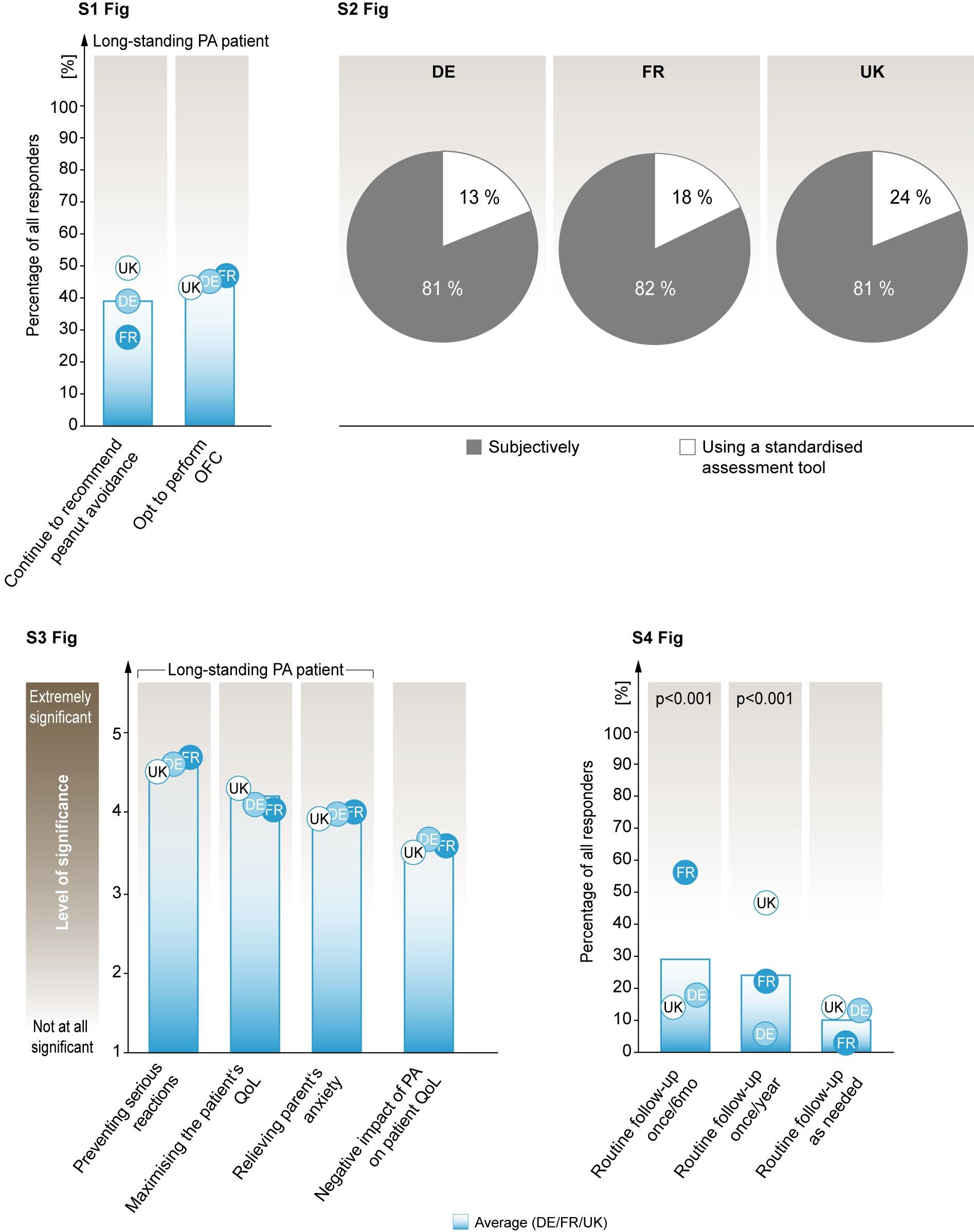

Supplement: S1 File — (TIF) [file pone.0241648.s001.tif]
