# Supplementary material for: A study to assess current approaches of allergists in European countries diagnosing and managing children and adolescents with peanut allergy
Source: PLoS One. 2020 Dec 3;15(12):e0241648. doi: 10.1371/journal.pone.0241648 (PMC7714149; doi:10.1371/journal.pone.0241648)
Supplement: S1 Table — (DOCX) [file pone.0241648.s002.docx]

**S1 Table. Supplementary results.**

|  | DE  (N=38) | FR  (N=36) | UK  (N=35) | EUR (N=109) | p-value  (3-way comparison) |
| --- | --- | --- | --- | --- | --- |
| *Screening Questions* |  |  |  |  |  |
| **Approximately how many patients do you manage for peanut allergy each month?** | 15 | 12 | 16 | 14 | 0.295 |
| **Approximately how many patients <18 years old do you manage for peanut allergy each month?** | 9 | 9 | 12 | 10 | 0.25 |
|  |  |  |  |  |  |
| **What is your primary practice setting?** |  |  |  |  |  |
| Community-based (most patients are seen in a private practice/community hospital) | 71% | 89% | 34% | 65% | <0.001 |
| Academic-based (most patients are seen in a university/academic hospital) | 29% | 11% | 66% | 35% | <0.001 |
|  |  |  |  |  |  |
| *Case #1* |  |  |  |  |  |
| **1. What testing would you perform to confirm a diagnosis at this time? (select all that apply)** | | | | | |
| Allergen-specific IgE | 95% | 86% | 49% | 77% | <0.001 |
| Allergen-specific IgG4 | 16% | 6% | 6% | 9% | 0.216 |
| Intradermal test | 13% | 19% | 6% | 13% | 0.224 |
| Oral food challenge | 13% | 14% | 3% | 10% | 0.225 |
| Peanut component testing (eg, IgE to Ara h 1, 2, 3) | 71% | 50% | 31% | 51% | 0.003 |
| Skin prick test | 61% | 86% | 66% | 71% | 0.04 |
| Total serum IgE | 63% | 31% | 34% | 43% | 0.008 |
| Other | 3% | 0% | 0% | 1% | 0.390 |
| I would not perform any testing in this patient | 0% | 6% | 14% | 7% | 0.044 |
| **2. Which of these (with or without the help of your staff) would you discuss with the patient and his mother at this visit or a follow-up visit? (select one for each item)** | | | | | |
| **Recognition of acute reactions** | | | | | 0.487 |
| Refer to a dietician or other healthcare provider to discuss | 5% | 3% | 11% | 6% | 0.310 |
| I would discuss at this visit | 92% | 97% | 86% | 92% | 0.211 |
| I would discuss at a later visit | 3% | 0% | 3% | 2% | 0.604 |
| I would not routinely discuss | 0% | 0% | 0% | 0% | - |
| **Use of adrenaline auto-injector** | | | | | 0.086 |
| Refer to a dietician or other healthcare provider to discuss | 0% | 0% | 6% | 2% | 0.116 |
| I would discuss at this visit | 79% | 81% | 77% | 79% | 0.94 |
| I would discuss at a later visit | 16% | 19% | 6% | 14% | 0.221 |
| I would not routinely discuss | 5% | 0% | 11% | 5% | 0.107 |
| **Allergen avoidance** | | | | | 0.676 |
| Refer to a dietician or other healthcare provider to discuss | 5% | 6% | 11% | 7% | 0.530 |
| I would discuss at this visit | 92% | 94% | 86% | 91% | 0.419 |
| I would discuss at a later visit | 3% | 0% | 3% | 2% | 0.604 |
| I would not routinely discuss | 0% | 0% | 0% | 0% | - |
| **Interpreting ingredient lists and food labels** | | | | | 0.358 |
| Refer to a dietician or other healthcare provider to discuss | 29% | 14% | 34% | 26% | 0.123 |
| I would discuss at this visit | 66% | 78% | 60% | 68% | 0.26 |
| I would discuss at a later visit | 3% | 8% | 3% | 5% | 0.422 |
| I would not routinely discuss | 3% | 0% | 3% | 2% | 0.604 |
| **Investigational therapies** |  |  |  |  | 0.025 |
| Refer to a dietician or other healthcare provider to discuss | 0% | 0% | 6% | 2% | 0.116 |
| I would discuss at this visit | 26% | 8% | 34% | 23% | 0.028 |
| I would discuss at a later visit | 37% | 44% | 17% | 33% | 0.042 |
| I would not routinely discuss | 37% | 47% | 43% | 42% | 0.662 |
| **Peanut allergy impact on quality of life** | | | | | 0.267 |
| Refer to a dietician or other healthcare provider to discuss | 5% | 0% | 6% | 4% | 0.357 |
| I would discuss at this visit | 61% | 61% | 63% | 62% | 0.978 |
| I would discuss at a later visit | 24% | 39% | 23% | 29% | 0.236 |
| I would not routinely discuss | 11% | 0% | 9% | 7% | 0.149 |
| **Prognosis** | | | | | 0.116 |
| Refer to a dietician or other healthcare provider to discuss | 0% | 0% | 0% | 0% | - |
| I would discuss at this visit | 76% | 56% | 83% | 72% | 0.028 |
| I would discuss at a later visit | 21% | 36% | 14% | 24% | 0.086 |
| I would not routinely discuss | 3% | 8% | 3% | 5% | 0.422 |
| **3. After his family becomes comfortable with food allergy management, approximately how frequently would you routinely see this patient back for follow-up? (select only one)** | | | | | <0.001 |
| Monthly | 11% | 0% | 6% | 6% | 0.139 |
| Every 3 months | 53% | 19% | 17% | 30% | 0.001 |
| Every 6 months | 18% | 56% | 14% | 29% | <0.001 |
| Yearly | 5% | 22% | 46% | 24% | <0.001 |
| Follow-up only as needed | 13% | 3% | 14% | 10% | 0.202 |
| Other | 0% | 0% | 3% | 1% | 0.344 |
| **4. Would you re-test this patient for peanut allergy at a later date to monitor for peanut allergy resolution? (select only one)** | | | | | 0.251 |
| Yes | 58% | 61% | 69% | 63% | 0.631 |
| No | 16% | 28% | 11% | 18% | 0.181 |
| Unsure | 26% | 11% | 20% | 19% | 0.251 |
| **4a. If this patient’s peanut allergy resolved, which recommendation would you make regarding peanut consumption and exposure? (select only one)** | | | | | 0.459 |
| Continue to avoid any peanut exposure to the extent possible | 50% | 23% | 25% | 33% | 0.098 |
| Eat only small servings of peanut on rare occasion | 9% | 18% | 13% | 13% | 0.667 |
| Ingest a normal serving of peanut regularly | 36% | 45% | 54% | 45% | 0.48 |
| Other | 5% | 14% | 8% | 9% | 0.565 |
|  |  |  |  |  |  |
| **5. When would you re-evaluate this patient for peanut allergy? (select only one)** | | | | | 0.623 |
| Every six months | 8% | 6% | 9% | 8% | 0.865 |
| Yearly | 33% | 50% | 26% | 36% | 0.093 |
| Every 2-3 years | 31% | 25% | 34% | 30% | 0.690 |
| Other (please specify) | 6% | 3% | 0% | 3% | 0.366 |
| I would not re-test this patient for peanut allergy | 14% | 14% | 23% | 17% | 0.508 |
| Unsure | 8% | 3% | 9% | 7% | 0.533 |
|  |  |  |  |  |  |
| *Case #2* |  |  |  |  |  |
| **6. Which of the following would you perform at this time? (select all that apply)** | | | | | |
| Assessment of nutritional status | 42% | 33% | 26% | 34% | 0.334 |
| Skin prick test | 61% | 72% | 60% | 64% | 0.472 |
| Total serum IgE | 39% | 22% | 23% | 28% | 0.174 |
| Other (please specify) | 0% | 0% | 3% | 1% | 0.344 |
| Assessment of quality of life | 42% | 47% | 60% | 50% | 0.294 |
| Renewal or revision of emergency action plan | 66% | 58% | 71% | 65% | 0.509 |
| Reinforcement of previous education about peanut allergy management | 58% | 50% | 83% | 64% | 0.011 |
| Allergen-specific IgE | 66% | 78% | 46% | 63% | 0.018 |
| Allergen-specific IgG4 | 13% | 11% | 11% | 12% | 0.958 |
| Intradermal test | 13% | 17% | 9% | 13% | 0.593 |
| Oral food challenge | 37% | 42% | 17% | 32% | 0.064 |
| Peanut component testing (eg, IgE to Ara h 1, 2, 3) | 53% | 58% | 34% | 48% | 0.106 |
| **6a. If “Assessment of quality of life” is selected above> How would you assess this patient’s quality of life? (select all that apply)** | | | | | |
| Subjectively | 81% | 82% | 81% | 81% | >0.99 |
| Using a standardized assessment tool (please specify which tool) | 13% | 18% | 24% | 18% | 0.676 |
| Other (please describe) | 0% | 0% | 0% | 0% | - |
| **7. How significant are each of the following goals in managing this patient? (select one for each item)** | | | | | |
| Prevent serious reactions | 4.55 | 4.67 | 4.51 | 4.58 | 0.674 |
| Maximizing the patient’s quality of life | 4.11 | 4.08 | 4.29 | 4.16 | 0.542 |
| Relieving the parent’s anxiety | 3.95 | 4.03 | 3.94 | 3.97 | 0.89 |
|  | | | | | |
| *Case #2 continued: You decide to repeat peanut allergy testing. The patient has specific IgE 1.5 kU/L and peanut skin prick test wheal 5 mm.* | | | | | |
| **8. How would you manage the patient now? (select only one)** | | | | | 0.132 |
| Continue to recommend avoidance without further testing at this time | 39% | 28% | 49% | 39% | 0.196 |
| Oral food challenge | 45% | 47% | 43% | 45% | 0.933 |
| Oral immunotherapy | 13% | 22% | 3% | 13% | 0.051 |
| Try small amount of peanut at home | 3% | 3% | 0% | 2% | 0.617 |
| Other | 0% | 0% | 6% | 2% | 0.116 |
| **9. When considering conducting an oral peanut challenge, which factors do you use in determining whether to conduct an oral food challenge? (select all that apply)** | | | | | |
| Patient age | 63% | 69% | 69% | 67% | 0.823 |
| History of acute reactions | 84% | 67% | 83% | 78% | 0.133 |
| Allergen-specific IgE | 68% | 69% | 63% | 67% | 0.817 |
| Skin prick results | 66% | 58% | 83% | 69% | 0.074 |
| Patient or parent desire for oral food challenge | 26% | 58% | 77% | 54% | <0.001 |
| I do not conduct oral food challenges in my patients | 21% | 19% | 3% | 14% | 0.055 |
| Other | 0% | 0% | 0% | 0% | - |
| **10. Do you typically include other healthcare professionals (eg, dietitians, social workers) in your management of patients with peanut allergy? (select only one)** | | | | | 0.294 |
| Yes | 63% | 61% | 77% | 67% |  |
| No | 37% | 39% | 23% | 33% |  |
| **10a. Which clinicians or specialists do you typically refer patients to for peanut allergy management? (select all that apply)** | | | | | |
| Dietitian or nutritionist | 92% | 82% | 96% | 90% | 0.103 |
| Social worker | 8% | 5% | 7% | 7% | 0.814 |
| Nurse educator | 17% | 14% | 74% | 35% | <0.001 |
| Psychologist or therapist | 8% | 27% | 11% | 15% | 0.249 |
| Other | 4% | 18% | 4% | 9% | 0.197 |
| **11. Proprietary information** | | | | | |
| **12. Proprietary information** | | | | | |
| **13. Please indicate the significance of each of the following barriers to the optimal management of patients with peanut allergy: (select one for each item)** | | | | | |
| Lack of effective treatments other than avoidance | 3.84 | 3.89 | 3.63 | 3.79 | 0.308 |
| Ubiquity of peanut in patients’ environments | 3.82 | 4.17 | 3.54 | 3.84 | 0.007 |
| Misconceptions or myths about peanut allergy | 3.42 | 3.89 | 3.54 | 3.62 | 0.069 |
| Patient bullying or harassment | 2.53 | 3 | 2.71 | 2.75 | 0.139 |
| Lack of time during patient visits | 3.29 | 3.33 | 2.97 | 3.20 | 0.224 |
| **14. In general, how challenging is allergen avoidance for your patients with peanut allergy? (select one)** | 3.58 | 3.11 | 3.46 | 3.38 | 0.041 |
| **15. To what extent does the peanut allergy negatively impact quality of life in your patients? (select one)** | 3.66 | 3.56 | 3.54 | 3.59 | 0.75 |
| **16. How do you prefer to make a final treatment decision for patients with peanut allergy? (select only one)** | | | | | 0.082 |
| I prefer to make the final decision about which treatment my patients receive | 32% | 19% | 11% | 21% | 0.104 |
| I prefer to make the final decision after seriously considering the patient’s/parent’s opinion | 34% | 14% | 34% | 27% | 0.082 |
| I prefer that the patient/parent and I share responsibility for deciding which treatment is best | 29% | 58% | 46% | 44% | 0.038 |
| I prefer that the patient/parent make the final decision, but after seriously considering my opinion | 5% | 8% | 9% | 7% | 0.831 |
| I prefer to leave all decisions regarding treatment to the patient/parent | 0% | 0% | 0% | 0% | - |
| **17. In well-controlled clinical trials, the following treatments demonstrated a 100-fold increase from baseline in the median tolerated dose after 12 months of treatment: (select one)** | | | | | |
| **Sublingual peanut immunotherapy** | | | | | 0.52 |
| TRUE | 37% | 28% | 20% | 28% | 0.279 |
| FALSE | 16% | 14% | 23% | 18% | 0.577 |
| Unsure | 47% | 58% | 57% | 54% | 0.582 |
| **Epicutaneous peanut immunotherapy** | | | | | 0.248 |
| TRUE | 16% | 25% | 17% | 19% | 0.561 |
| FALSE | 45% | 31% | 23% | 33% | 0.129 |
| Unsure | 39% | 44% | 60% | 48% | 0.191 |
| **Oral peanut immunotherapy** | | | | | 0.315 |
| TRUE | 37% | 42% | 54% | 44% | 0.305 |
| FALSE | 18% | 8% | 6% | 11% | 0.183 |
| Unsure | 45% | 50% | 40% | 45% | 0.698 |
| **18. Data from different clinical trials suggest that the following treatments for peanut allergy are associated with similar rates of adverse reactions as oral immunotherapy: (select one)** | | | | | |
| **Sublingual immunotherapy** | | | | | 0.688 |
| TRUE | 42% | 36% | 31% | 36% | 0.637 |
| FALSE | 11% | 17% | 23% | 17% | 0.366 |
| Unsure | 47% | 47% | 46% | 47% | 0.988 |
| **Epicutaneous immunotherapy** | | | | | 0.723 |
| TRUE | 16% | 11% | 23% | 17% | 0.407 |
| FALSE | 34% | 39% | 29% | 34% | 0.656 |
| Unsure | 50% | 50% | 49% | 50% | 0.99 |
| **19. Please rate your familiarity with the following emerging therapies for peanut allergy: (select one for each item)** | | | | | |
| AR101 oral immunotherapy | 2.42 | 2.17 | 2.40 | 2.33 | 0.532 |
| Peanut subcutaneous immunotherapy | 2.00 | 2.25 | 1.91 | 2.05 | 0.396 |
| Peanut sublingual immunotherapy | 2.26 | 2.39 | 2.03 | 2.23 | 0.363 |
| Peanut epicutaneous immunotherapy | 1.97 | 2.50 | 2.09 | 2.19 | 0.09 |
| **20. If you were considering oral immunotherapy for a patient with peanut allergy, rank the following factors order of importance in your decision of whether to recommend oral immunotherapy? (Rank 1 to 5)** | | | | | |
| Severity of peanut allergy | 4.45 | 4.44 | 4.26 | 4.38 | 0.678 |
| Patient/caregiver desire to undergo immunotherapy | 3.03 | 3.19 | 2.97 | 3.06 | 0.701 |
| Patient/caregiver concerns about quality of life | 2.50 | 2.83 | 3.20 | 2.84 | 0.024 |
| Patient age | 3.13 | 2.92 | 3.06 | 3.04 | 0.727 |
| Insurance coverage for oral immunotherapy | 1.89 | 1.61 | 1.51 | 1.67 | 0.357 |
| **21. If multiple immunotherapies for peanut allergy become available, how significant will the following factors be in selecting between treatments? (select one for each item)** | | | | | |
| Data supporting efficacy | 4.29 | 4.64 | 4.31 | 4.41 | 0.07 |
| Safety profile | 4.42 | 4.47 | 4.49 | 4.46 | 0.928 |
| Potential for loss of desensitization when therapy is discontinued | 3.76 | 3.92 | 4.03 | 3.90 | 0.38 |
| Scheduling burden and time required for treatments | 3.18 | 3.75 | 3.89 | 3.61 | 0.001 |
| Cost or insurance coverage | 3.21 | 3.28 | 2.94 | 3.14 | 0.405 |
| Convenience | 3.61 | 3.69 | 3.60 | 3.63 | 0.828 |
| Ability to assess patient response | 3.61 | 4.00 | 4.03 | 3.88 | 0.051 |
| **22. How important are the following factors in improving your comfort level if you are considering implementing a new drug or treatment? (select one for each item)** | | | | | |
| Efficacy data from clinical trials | 4.24 | 4.47 | 4.37 | 4.36 | 0.439 |
| Safety data from clinical trials | 4.42 | 4.42 | 4.57 | 4.47 | 0.562 |
| Real world data | 3.74 | 4.28 | 4.20 | 4.07 | 0.014 |
| FDA approval | 4.11 | 4.08 | 4.03 | 4.07 | 0.935 |
| Inclusion of treatment in nationally recognized treatment guideline | 3.97 | 3.97 | 4.34 | 4.09 | 0.078 |
| **23. How concerned are you about each of the following as it relates to investigational oral immunotherapies for peanut allergy? (select one for each item)** | | | | | |
| Risk of adverse effects | 3.61 | 3.94 | 4.20 | 3.92 | 0.01 |
| The need for maintenance dosing | 2.92 | 3.42 | 3.57 | 3.30 | 0.002 |
| Lack of efficacy | 3.26 | 3.72 | 3.89 | 3.62 | 0.025 |
| Patient reluctance to undergo immunotherapy | 2.76 | 3.58 | 3.66 | 3.33 | <0.001 |
| Patient lack of adherence to immunotherapy treatment | 3.34 | 4.03 | 3.97 | 3.78 | 0.001 |
| Lack of data supporting long-term outcomes | 3.39 | 4.00 | 4.06 | 3.82 | 0.001 |
| Logistics of administering therapy | 2.84 | 3.47 | 3.57 | 3.29 | <0.001 |
| **24. Do you administer unregulated (eg, “home brew”) oral immunotherapies to your patients with peanut allergy? (select only one)** | | | | | 0.464 |
| Yes | 8% | 14% | 6% | 9% |  |
| No | 92% | 86% | 94% | 91% |  |
| **25. Practice location: (select only one)** | | | | | 0.705 |
| Urban | 74% | 78% | 80% | 77% | 0.808 |
| Suburban | 16% | 8% | 14% | 13% | 0.602 |
| Rural | 11% | 14% | 6% | 10% | 0.517 |
