# Supplementary material for: A study to assess current approaches of allergists in European countries diagnosing and managing children and adolescents with peanut allergy
Source: PLoS One. 2020 Dec 3;15(12):e0241648. doi: 10.1371/journal.pone.0241648 (PMC7714149; doi:10.1371/journal.pone.0241648)
Supplement: S2 Table — (DOCX) [file pone.0241648.s003.docx]

**S2 Table. A list of all survey questions.**

**Screening Questions**

**Approximately how many patients do you manage for peanut allergy each month?** <if 0, screen out>

**Approximately how many patients <18 years old do you manage for peanut allergy each month?** <if 0, screen out>

**What is your primary practice setting?** (select only one)

| ❑ | Community-based (most patients are seen in a private practice/community hospital) |
| --- | --- |
| ❑ | Academic-based (most patients are seen in a university/academic hospital) |

**Case-vignette Questions**

**Case #1**: A 2-year-old boy presents with his mother for a peanut allergy evaluation. Several weeks ago, he had eaten about 1 teaspoon (5 mL) of peanut butter. Within a few minutes, he developed several “hives” on his face and torso with pruritus without other concerning symptoms such as lip or tongue swelling, coughing, difficulty breathing, or vomiting. After calling his pediatrician’s office, he was given diphenhydramine with resolution of symptoms. His family does not recall any prior peanut exposures. He does not have any significant allergy history as other food allergies, atopic dermatitis, or wheezing episodes.

**What testing would you perform to confirm a diagnosis at this time?** (select all that apply)

| ❑ | Allergen-specific IgE |
| --- | --- |
| ❑ | Allergen-specific IgG4 |
| ❑ | Intradermal test |
| ❑ | Oral food challenge |
| ❑ | Peanut component testing (eg, IgE to Ara h 1, 2, 3) |
| ❑ | Skin prick test |
| ❑ | Total serum IgE |
| ❑ | Other (please specify) |
| ❑ | I would not perform any testing in this patient <exclusive answer option> |

**Case #1 continued**: The boy undergoes skin prick testing with appropriate positive and negative controls. His peanut skin test reveals a 7 mm wheal. Peanut specific IgE is 1.9 kU/L.

**Which of these (with or without the help of your staff) would you discuss with the patient and his mother at this visit or a follow-up visit?** (select one for each item)

|  | **Refer to a dietician or other healthcare provider to discuss** | **I would discuss at this visit** | **I would discuss at a later visit** | **I would not routinely discuss** |
| --- | --- | --- | --- | --- |
| Recognition of acute reactions | ❑ | ❑ | ❑ | ❑ |
| Use of adrenaline auto-injector | ❑ | ❑ | ❑ | ❑ |
| Allergen avoidance | ❑ | ❑ | ❑ | ❑ |
| Interpreting ingredient lists and food labels | ❑ | ❑ | ❑ | ❑ |
| Investigational therapies | ❑ | ❑ | ❑ | ❑ |
| Peanut allergy impact on quality of life | ❑ | ❑ | ❑ | ❑ |
| Prognosis | ❑ | ❑ | ❑ | ❑ |

**After his family becomes comfortable with food allergy management, approximately how frequently would you routinely see this patient back for follow-up?** (select only one)

| ❑ | Monthly |
| --- | --- |
| ❑ | Every 3 months |
| ❑ | Every 6 months |
| ❑ | Yearly |
| ❑ | Follow-up only as needed |
| ❑ | Other (please specify) |

**Would you re-test this patient for peanut allergy at a later date to monitor for peanut allergy resolution?** (select only one)

| ❑ | Yes |
| --- | --- |
| ❑ | No |
| ❑ | Unsure |

- 1. <If “Yes” selected above> **If this patient’s peanut allergy resolved, which recommendation would you make regarding peanut consumption and exposure?** (select only one)

| ❑ | Continue to avoid any peanut exposure to the extent possible |
| --- | --- |
| ❑ | Eat only small servings of peanut on rare occasion |
| ❑ | Ingest a normal serving of peanut regularly |
| ❑ | Other (please specify) |

**When would you re-evaluate this patient for peanut allergy?** (select only one)

| ❑ | Every six months |
| --- | --- |
| ❑ | Yearly |
| ❑ | Every 2-3 years |
| ❑ | Other (please specify) |
| ❑ | I would not re-test this patient for peanut allergy <exclusive answer option> |
| ❑ | Unsure |

**Case #2**: A 12-year old boy comes to your office with his parents. He has a long-standing peanut allergy diagnosis. His last allergy testing occurred several years ago with a peanut specific IgE 0.9 kU/L. At that time, he underwent a challenge and developed hives after consuming ~300 mg peanut protein (approximately 1 peanut). For the last year, he has become increasing frustrated in dealing with his allergy. He admits to being less careful about food allergy management. He often does not carry an adrenaline autoinjector or read labels before eating foods. He describes taking a small bite of a food to see if it is safe. He has not experienced any recent reactions so feels that his approach to his peanut allergy is “good enough,” but his parents are concerned about his attitude toward his allergy.

His medical history is notable for tree nut allergy and moderate persistent asthma. He has poor adherence to his prescribed asthma medications and has had two exacerbations in the past year requiring systemic corticosteroids.

**Which of the following would you perform at this time?** (select all that apply)

| ❑ | Assessment of nutritional status |
| --- | --- |
| ❑ | Assessment of quality of life |
| ❑ | Renewal or revision of emergency action plan |
| ❑ | Reinforcement of previous education about peanut allergy management |
| ❑ | Allergen-specific IgE |
| ❑ | Allergen-specific IgG4 |
| ❑ | Intradermal test |
| ❑ | Oral food challenge |
| ❑ | Peanut component testing (eg, IgE to Ara h 1, 2, 3) |
| ❑ | Skin prick test |
| ❑ | Total serum IgE |
| ❑ | Other (please specify) |

- 1. <If “Assessment of quality of life” is selected above> **How would you assess this patient’s quality of life?** (select all that apply)

| ❑ | Subjectively |
| --- | --- |
| ❑ | Using a standardized assessment tool (eg, Food Allergy Quality of Life Questionnaire, SF-36) (please specify which tool) |
| ❑ | Other (please describe) |

**How significant are each of the following goals in managing this patient?** (select one for each item)

|  | Not at all significant | Slightly significant | Moderately significant | Very significant | Extremely significant |
| --- | --- | --- | --- | --- | --- |
| Prevent serious reactions | ❑ | ❑ | ❑ | ❑ | ❑ |
| Maximizing the patient’s quality of life | ❑ | ❑ | ❑ | ❑ | ❑ |
| Relieving the parent’s anxiety | ❑ | ❑ | ❑ | ❑ | ❑ |

**Case #2 continued**: You decide to repeat peanut allergy testing. The patient has specific IgE 1.5 kU/L and peanut skin prick test wheal 5 mm.

**How would you manage the patient now?** (select only one)

| ❑ | Continue to recommend avoidance without further testing at this time |
| --- | --- |
| ❑ | Oral food challenge |
| ❑ | Oral immunotherapy |
| ❑ | Try small amount of peanut at home |
| ❑ | Other (please specify) |

**Non-case Questions**

**When considering conducting an oral peanut challenge, which factors do you use in determining whether to conduct an oral food challenge?** (select all that apply)

| ❑ | Patient age |
| --- | --- |
| ❑ | History of acute reactions |
| ❑ | Allergen-specific IgE |
| ❑ | Skin prick results |
| ❑ | Patient or parent desire for oral food challenge |
| ❑ | I do not conduct oral food challenges in my patients |
| ❑ | Other (please specify) |

**Do you typically include other healthcare professionals (eg, dietitians, social workers) in your management of patients with peanut allergy?** (select only one)

| ❑ | Yes |
| --- | --- |
| ❑ | No |

- 1. <If “Yes” is selected above> **Which clinicians or specialists do you typically refer patients to for peanut allergy management?** (select all that apply)

| ❑ | Dietitian or nutritionist |
| --- | --- |
| ❑ | Social worker |
| ❑ | Nurse educator |
| ❑ | Psychologist or therapist |
| ❑ | Other (please specify) |

**Proprietary information**

**Proprietary information**

**Please indicate the significance of each of the following barriers to the optimal management of patients with peanut allergy:** (select one for each item)

|  | Not applicable or not at all significant | Slightly significant | Moderately significant | Very significant | Extremely significant |
| --- | --- | --- | --- | --- | --- |
| Lack of effective treatments other than avoidance | ❑ | ❑ | ❑ | ❑ | ❑ |
| Ubiquity of peanut in patients’ environments | ❑ | ❑ | ❑ | ❑ | ❑ |
| Misconceptions or myths about peanut allergy | ❑ | ❑ | ❑ | ❑ | ❑ |
| Patient bullying or harassment | ❑ | ❑ | ❑ | ❑ | ❑ |
| Lack of time during patient visits | ❑ | ❑ | ❑ | ❑ | ❑ |

**In general, how challenging is allergen avoidance for your patients with peanut allergy?** (select one)

| Not at all challenging | Slightly challenging | Moderately challenging | Very challenging | Extremely  challenging |
| --- | --- | --- | --- | --- |
| ❑ | ❑ | ❑ | ❑ | ❑ |

**To what extent does the peanut allergy negatively impact quality of life in your patients?** (select one)

| Not at all | Slightly | Moderately | Very | Extremely |
| --- | --- | --- | --- | --- |
| ❑ | ❑ | ❑ | ❑ | ❑ |

**how do you prefer to make a final treatment decision for patients with peanut allergy?** (select only one)

| ❑ | I prefer to make the final decision about which treatment my patients receive |
| --- | --- |
| ❑ | I prefer to make the final decision after seriously considering the patient’s/parent’s opinion |
| ❑ | I prefer that the patient/parent and I share responsibility for deciding which treatment is best |
| ❑ | I prefer that the patient/parent make the final decision, but after seriously considering my opinion |
| ❑ | I prefer to leave all decisions regarding treatment to the patient/parent |

**In well-controlled clinical trials, the following treatments demonstrated a 100-fold increase from baseline in the median tolerated dose after 12 months of treatment:** (select one for each item)

|  | True | False | Unsure |
| --- | --- | --- | --- |
| Sublingual peanut immunotherapy | ❑ | ❑ | ❑ |
| Epicutaneous peanut immunotherapy | ❑ | ❑ | ❑ |
| Oral peanut immunotherapy | ❑ | ❑ | ❑ |

**Data from different clinical trials suggest that the following treatments for peanut allergy are associated with similar rates of adverse reactions as oral immunotherapy:** (select one for each item)

|  | True | False | Unsure |
| --- | --- | --- | --- |
| Sublingual immunotherapy | ❑ | ❑ | ❑ |
| Epicutaneous immunotherapy | ❑ | ❑ | ❑ |

**Please rate your familiarity with the following emerging therapies for peanut allergy:** (select one for each item)

|  | Not at all familiar | Slightly familiar | Moderately familiar | Very familiar | Extremely familiar |
| --- | --- | --- | --- | --- | --- |
| AR101 oral immunotherapy | ❑ | ❑ | ❑ | ❑ | ❑ |
| Peanut subcutaneous immunotherapy | ❑ | ❑ | ❑ | ❑ | ❑ |
| Peanut sublingual immunotherapy | ❑ | ❑ | ❑ | ❑ | ❑ |
| Peanut epicutaneous immunotherapy | ❑ | ❑ | ❑ | ❑ | ❑ |

**If you were considering oral immunotherapy for a patient with peanut allergy, how would you rank the following factors in order of importance in your decision of whether to recommend oral immunotherapy?** (Rank 1 to 5 with 1 being the most important and 5 being the least important factor)

|  | Severity of peanut allergy |
| --- | --- |
|  | Patient/caregiver desire to undergo immunotherapy |
|  | Patient/caregiver concerns about quality of life |
|  | Patient age |
|  | Insurance coverage for oral immunotherapy |

**If multiple immunotherapies for peanut allergy become available, how significant will the following factors be in selecting between treatments?** (select one for each item)

|  | Not at all significant | Slightly significant | Moderately significant | Very significant | Extremely significant |
| --- | --- | --- | --- | --- | --- |
| Data supporting efficacy | ❑ | ❑ | ❑ | ❑ | ❑ |
| Safety profile | ❑ | ❑ | ❑ | ❑ | ❑ |
| Potential for loss of desensitization when therapy is discontinued | ❑ | ❑ | ❑ | ❑ | ❑ |
| Scheduling burden and time required for treatments | ❑ | ❑ | ❑ | ❑ | ❑ |
| Cost or insurance coverage | ❑ | ❑ | ❑ | ❑ | ❑ |
| Convenience | ❑ | ❑ | ❑ | ❑ | ❑ |
| Ability to assess patient response | ❑ | ❑ | ❑ | ❑ | ❑ |

**How important are the following factors in improving your comfort level if you are considering implementing a new drug or treatment?** (select one for each item)

|  | Not at all important | Slightly important | Moderately important | Very important | Extremely important |
| --- | --- | --- | --- | --- | --- |
| Efficacy data from clinical trials | ❑ | ❑ | ❑ | ❑ | ❑ |
| Safety data from clinical trials | ❑ | ❑ | ❑ | ❑ | ❑ |
| Real world data | ❑ | ❑ | ❑ | ❑ | ❑ |
| FDA approval | ❑ | ❑ | ❑ | ❑ | ❑ |
| Inclusion of treatment in nationally recognized treatment guideline | ❑ | ❑ | ❑ | ❑ | ❑ |

**How concerned are you about each of the following as it relates to investigational oral immunotherapies for peanut allergy?** (select one for each item)

|  | Not at all concerned | Slightly concerned | Moderately concerned | Very concerned | Extremely concerned |
| --- | --- | --- | --- | --- | --- |
| Risk of adverse effects | ❑ | ❑ | ❑ | ❑ | ❑ |
| The need for maintenance dosing | ❑ | ❑ | ❑ | ❑ | ❑ |
| Lack of efficacy | ❑ | ❑ | ❑ | ❑ | ❑ |
| Patient reluctance to undergo immunotherapy | ❑ | ❑ | ❑ | ❑ | ❑ |
| Patient lack of adherence to immunotherapy treatment | ❑ | ❑ | ❑ | ❑ | ❑ |
| Lack of data supporting long-term outcomes | ❑ | ❑ | ❑ | ❑ | ❑ |
| Logistics of administering therapy | ❑ | ❑ | ❑ | ❑ | ❑ |

**Do you administer <NOTE: “**non-FDA approved” for US “unregulated” for EU> **unregulated (eg, “home brew”) oral immunotherapies to your patients with peanut allergy?** (select only one)

| ❑ | Yes |
| --- | --- |
| ❑ | No |

**Practice location:** (select only one)

| ❑ | Urban |
| --- | --- |
| ❑ | Suburban |
| ❑ | Rural |
